# The 10 years’ experience in the laparoscopic treatment of benign pathology of the eso gastric junction

**Published:** 2012-06-18

**Authors:** L Lucenco, M Marincas, C Cirimbei, E Bratucu, S Ionescu

**Affiliations:** Gastroenterology Department, University Emergency Hospital, Bucharest

**Keywords:** GERD, cardiospasm, Nissen fundoplication, Toupet fundoplication, esocardiomiotomy

## Abstract

In the era of mini invasive surgery, the surgical approach of the esogastric junction occupies an important role, which regards both the results and the complete, long-term patient satisfaction.

The main benign pathology of the esogastric pole includes hiatal hernia, gastroesophageal reflux disease, cardiospasm, oesophageal diverticula. The present study is based on the experience of our clinic in the laparoscopic treatment of esogastric pathology that contains 85 patients in 10 years. Out of these, 15 were operated on for cardiospasm, 29 for hiatal hernia and 41 for gastroesophageal reflux disease (GERD). The investigation protocol consisted in barium swallows and endoscopy, both pre and postoperatively. The results obtained allowed us to underline the superiority of the surgical treatment over the medical one. Likewise, medical literature reports rates of success of 90% in antireflux surgery. The latter is conditioned by correct determination of the reflux causes and by the choice of the adequate time to perform the surgery, in concordance with the local anatomical conditions. As far as the two techniques used (complete or partial fundoplication) are concerned, there were no significant differences in the postoperative evolution of the patients, but we have to mention, nevertheless, the increased incidence of dysphagia after Nissen. The data presented confirm the superiority of laparoscopic surgery over the classic one, due to the superior aesthetic result, the shortened admission time –with reduced costs and rapid social reinsertion.

**Abbreviations**GERD – gastroesophageal reflux disease, LES – lower esophagian sphincter

## Introduction

The event that has marked the surgical world in the last two decades was the introduction of laparoscopic surgery, which gained more and more confidence compared to the classic surgery. After vesicular lithiasis, in which laparoscopic surgery became the main approach, ever since 1991 after the first Nissen was done laparoscopically, the mini invasive surgery of the esogastric junction became more important. The frequency of the gastroesophageal reflux pathology (it is supposed that between 15 and 40 % of the population has GERD symptoms at least once a month) and the incontestable benefits of the lap surgery (aesthetics, absence of pain, reduced admission costs, early mobilisation, rapid social insertion), both contributed to patients’ choice of mini invasive surgery. [**1**]

The main benign pathology of the esogastric pole, in which we used laparoscopic approach, is hiatal hernias, reflux gastroesophageal disease (GERD), cardiospasm, oesophageal diverticula. Out of these, the most frequent are GERD and hiatal hernias. The role of esocardial junction is to assure the passing of the food bole in one direction: gravitational, descendant, from the oesophagus to the duodenum. Out of the mechanisms which involve gastroesophageal reflux, we should mention the oesophageal peristalsis, the antireflux machine-composed of the diaphragm, the normal contraction of the oesophageal hiatus, the Laimer Bertelli freno-oesofageal membrane, the lower oesophageal sphincter LES, the sharp angle between the stomach and the oesophagus and the Gubaroff valve, the wash out effect of the saliva, the capacity of protection of the mucosa, the evacuation and deposit function of the stomach. The loss of any of those mechanisms inevitably leads to reflux. [**2**]

The cardial contention is assured by LES, which represents the primary antireflux barrier. The main pressure at the level of the thoracic oesophagus is negative (between+15 and +5 mmHg), reflecting the intra-pleural pressure, and the pressure in the stomach is positive (between +7 and +50). The pressure gradient which determines the acid reflux in the stomach is situated between +5 and +15mm Hg. LES is a physiologic sphincter, representing a high-pressure zone (HPZ) of 3-3,5cm, which maintains its basal tone above the intra-gastric pressure. LES was identified through oesophageal manometry, on the one side and on the other of the diaphragmatic hiatus (two thirds in the abdomen and one in the thorax). LES is normally closed and is relaxed during deglutition, eructation and vomit. The relaxation lasts for 6-9 sec. The prolongation of the relaxation time, by relaxation of the gastric body and the gastric fundus leads to the appearance of reflux. The tonus of LES is manometrically registered, being a predictive factor in the appreciation of the reflux type and the surgical technique to be applied. [**3**]

The gastroesophageal reflux (GER) is the involuntary pass of a certain part of the gastric content, which is produced without a sensation of vomit without a contraction of the gastric muscle or of the anterior abdominal wall, into the oesophagus. GER is a physiologic syndrome that expresses the insufficiency of the cardia and that of the LES. The reflux esophagitis is a syndrome characterized by inflammatory lesions of the oesophageal mucosa due to the repeated reflux of the chloro-peptical or bilio-pancreatic juices into the inferior oesophagus.

The mechanisms involved in GER are:

-the deficit of the antireflux barrier

-the prolongation of the oesophageal clearance

-the delayed gastric emptying

-the low capacity of defence of the oesophageal mucosa

-the hyperacid gastric secretion

From a clinical point of view, GER may be mild, medium, severe and advanced (peptic strictures). [2,3]

Another classification divides GER in primary reflux (after valve and sphincter deficit) and secondary reflux (in pathological states scleroderma, gastric atony, pyloric stenosis, after gastric resections with vagotomy).

A bigger GER incidence was found in women, the main age being of more than 50. According to De Meester, 80% of the patients with GER present hiatal hernia. [**4**]

The hiatal hernia represents the trans-diaphragmatic migration of the stomach from the abdominal cavity into the thorax, through the oesophageal hiatus. The oldest classification of the hiatal hernia dates from 1926 and belongs to Ackerlund:

-type I hiatal hernia through brachy-oesophagus, in which the cardia and the fornix are in the thorax

-type II axial hiatal hernia, in which the cardia and partially the fornix are above the diaphragm

-type III para-oesophageal hiatal hernia in which only one part of the fornix migrates into the thorax, the cardia remaining in place.

**Fig. 1:**
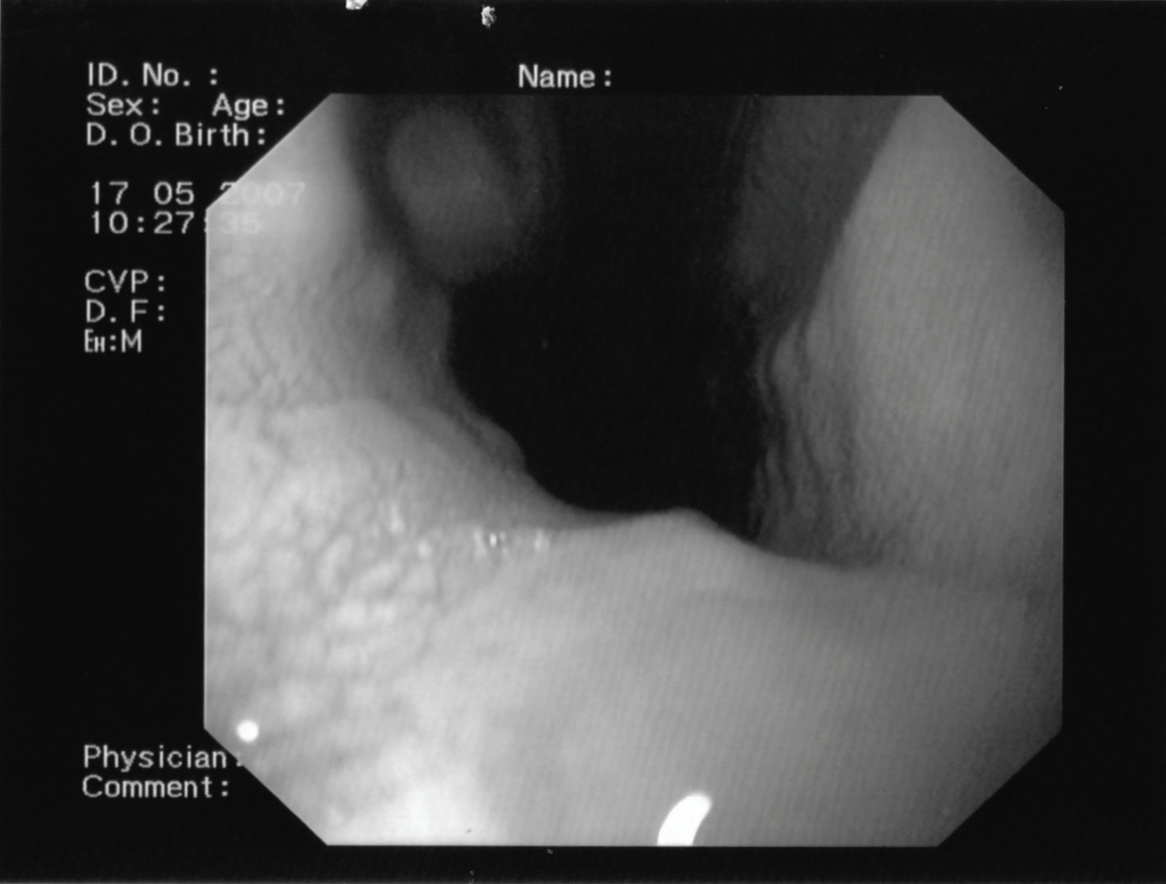
Endoscopy: a big hiatal hernia (6 cm diameter)

Another classification, by the means of appearance, was done by Sweet and Allison:

-type I sliding hiatal hernia, or axial (**Fig. 1**)

**Fig. 2:**
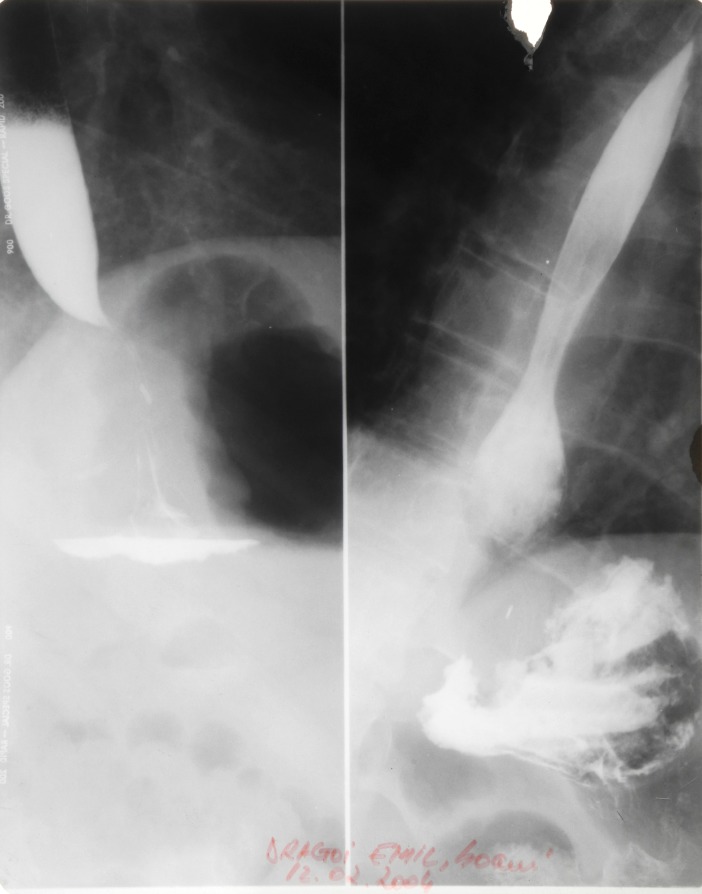
Barium swallow: a paraesophaegeal hernia

-type II rolling hiatal hernia (para-oesophageal), with peritoneal fold (**Fig. 2**)

-Type III mixt hiatal hernia

Out of the most frequent cases of hiatal hernia, we mention:

-obesity;

-vertebral column deformation which implies disfunctionality of the diaphragmatic pillars

-pregnancy due to the increase in progesterone and vomits

-endocrine factors

-smoking

-chronic constipation

-chronic cough

-trauma of the abdomen and thorax 

It was proved that 55% of the patients with hiatal hernia present GER. [**5**]

The clinical symptoms of hiatal hernia are:

1. typical symptoms

-pyrosis

-thoracic pain (sign of the reflux esophagitis)

-regurgitations

-dysphagia

-odynophagia

-sialorrhea 

2. GER symptoms

-pyrosis, eructation, pain in the epigastrium

-sometimes atypical symptoms: dysphonia, pharyngeal pain, cough, thoracic pain

-alarm signs, which appear in the complication phase: haemorrhage and dysphagia

3. atypical symptoms 

-ENT

-pulmonary

-asthma exacerbation

-angina

4. symptoms due to complications

-hematemesis or melena

-hypochromic anaemia

-signs of duodenal ulcer

-signs of neoplasm on Barrett oesophagus [**5,6**]

### Paraclinical exploration

Barium swallow is the first investigation to do in a patient with esogastric pathology, in which we exclude at the beginning other associated lesions or diseases. Several techniques are employed: monocontrast, double contrast in standard or with provocation of the reflux (Trendelenburg, Brombard), using Valsalva and Muller. Barium swallow offers images on the length, calibre and position of the oesophagus, the presence of the hiatal hernia and its type and also gives information on the stomach and its efficiency of evacuation. If the radiological examination is negative, one addresses the other means of investigation.

Endoscopy offers useful information on the esogastric anatomy and its modifications due to GER, the presence of hiatal hernia, allowing the appreciation of the grade of esophagitis, and also the ascension of the gastric mucosa in comparison to the hiatus.

The endoscopic classification of esophagitis lesions, after Savary and Miller:

-grade I (minimal esophagitis) superficial erythema of the distal oesophagus with capillary dilation

-Grade II (medium esophagitis) the loss of detail of the esogastric junction with discrete erosions of dark red colour, with a tendency to conglomerate.

**Fig. 3:**
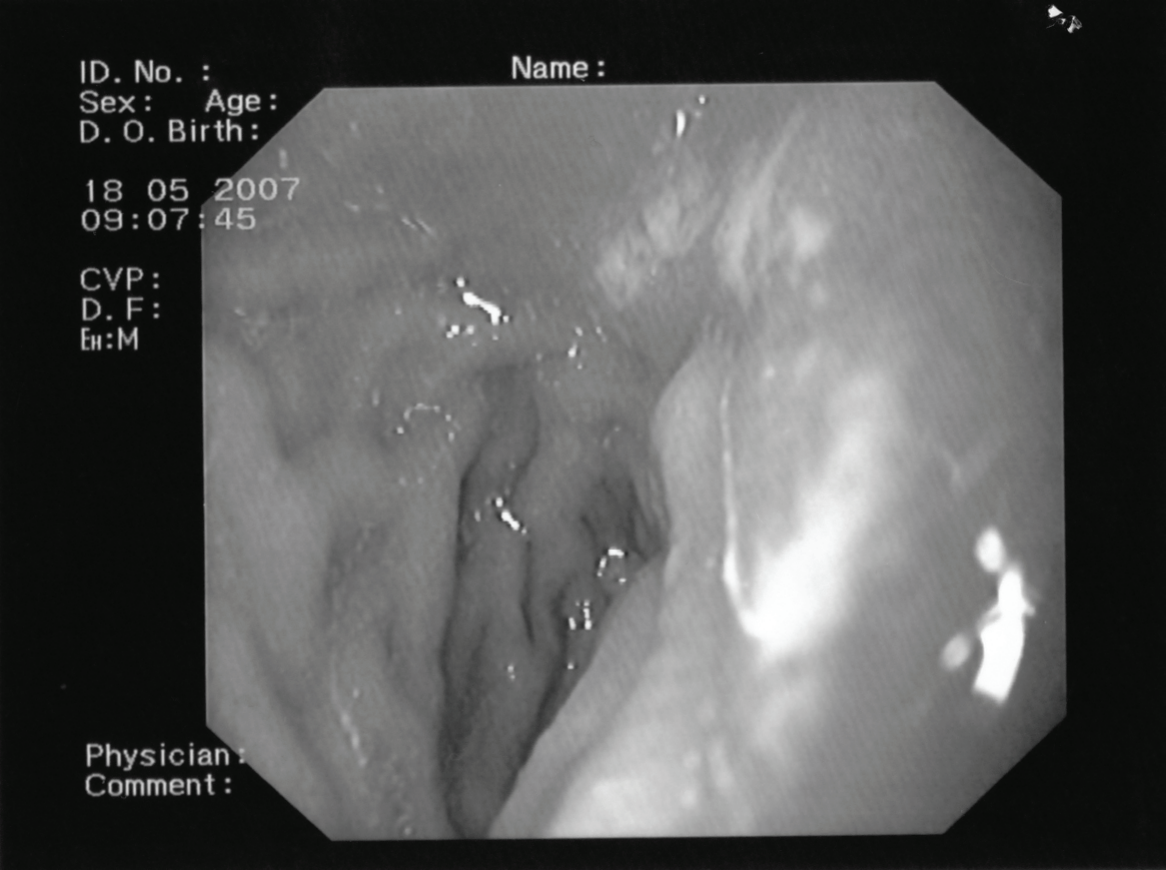
Endoscopy: severe esophagitis, circumferential ulcerations with granulation tissue of the oesophageal wall

-grade III (severe esophagitis) circumferential ulcerations with granulation tissue fibrosis of the oesophageal wall which limits its distension with insufflation (**Fig. 3**)

-grade IV (stricture) manifests similar to grade III at which stenosis of the oesophageal lumen is added with old lesions of the columnar epithelium and short oesophagus. [**7**]

If the GER lesions are not found endoscopically, one may recur to other explorations, such as monitored pH metry, the safest exploration to point to GER, and also the exposal of the oesophagus to reflux. The most often used is the 24-hour monitoring which allows the quantitative evaluation of the oesophageal exposure to gastric juice. The test is positive if the pH drops under 4, for more than 5 minutes. The probe is very sensitive, being used both pre and postoperatively. Monitored PH metry allows the determination of the number of the reflux period, of more than 5 minutes, the lasting of the longest reflux episode and the main duration of the reflux episodes with a pH under 4. [**2,3**]

Another useful test in the evaluation of the mechanic oesophageal dysfunction is the oesophageal manometry. The method is useful for the preoperative selection of the patients with mechanic deficits of the LES and the evaluation of the oesophageal peristalsis.

Standard manometry explores 4 elements:

-competence of the LES;

-relaxation of the LES;

-the peristalsis of the oesophageal wall

-competence of the upper oesophageal sphincter (cricopharyngeal)

Other explorations less accessible and trustable are oesophageal scintigraphy pass, reflux scintigraphy, the ambulatory monitoring of the upper digestive tract, videocineradiography, biochemistry of the gastric and oesophageal aspirate.

### Treatment principles

Medical treatment is the first treatment attack and it is sometimes enough when associated with diet measures (fat lowering in the diet, cut in the gaseous liquids, smoking prohibition, dieting). The strategy of the medical treatment involves the association of prokinetics (metoclopramide, cisapride, motilium) with protectors of the gastric mucosa (sucralfate) and anti H2 (famotidine, zantac) or inhibitors of the protonic pump (omeprazole, nexium).

The treatment may be initially led under the attack form, for 4-12 weeks, followed by a maintenance treatment, for one year. The attack treatment may be done either in a step up manner or regressively in a step down manner. [**1,5**]

The failure of the medical treatment or the apparition of complications indicates the surgical treatment. The indications of the surgical treatment are:

-failure of a medical treatment that was correctly implemented or the recurrence of symptoms after treatment interruption 

-presence of severe esophagitis which does not respond to medical treatment

-massive gastroesophageal reflux, without esophagitis, but with a high risk of pulmonary complications

-presence of Barret oesophagus

-peptic stenosis or brachy-oesophagus

-association of two types of hernia sliding and rolling or isolated para-oesophageal hernia

-patients that opt for surgical treatment due to lifestyle worsening after a prolonged medical treatment or out of socio economic reasons (expensive treatment) [**6**]

### Surgical treatment

As far as GERD is concerned, the technically applicable variations are:

-Nissen fundoplication, Nissen Rosetti and floppy Nissen

-Toupet fundoplication

-Dor fundoplication 

-Hill procedure

-round ligament cardiopexy

The success of the procedure is due to its correct indication. The applied technique will be conditioned by:

-documentation of the fact that LES is the cause of the reflux

-elimination of other reflux causes: the decrease in oesophageal clearance or in gastric emptying

-Objectivation through oesophageal manometry, esogastric endoscopy, pH metry, gastric evacuation scintigraphy and barium swallow.

Choosing the surgical procedure against reflux depends on the length of the oesophagus and on its motor function. Nissen fundoplication is indicated in cases in which the oesophagus is normal in length and with a normal motor function. [**8,9**]

Toupet fundoplication is recommended in cases with normal length but decreased motor function.

Through the thorax, one can perform the Belsey procedure with a valve of 270 degrees, recommended in cases of short oesophagus with a normal function.

In cases of hiatal hernia, the treatment’s success is conditioned by the correct and complete diagnosis, the correct reduction of the hernia from the mediastinum, the correct calibration of the hiatal hiatus (with or without a substitution prosthesis) and the performance of an antireflux procedure adjusted to the oesophageal function.

Heller esocardiomiomectomy is the most efficient treatment method in cardiospasm, but, due to the destruction of the LES, the complete sectioning of the muscle fibres favours the apparition of GERD. In order to avoid this inconvenient, a partial Toupet or Dor fundoplication is associated in order to maintain the sections separately. The treatment’s success is conditioned by the complete establishment of the diagnosis and the rigorous operatory technique –sectioning of the fibres for at least 6 cm and 2 cm on the cardia.

## Material and method

We performed a retrospective study on 85 patients with diseases of the esogastric pole treated between 2001 and 2011. 15 patients were operated on for cardiospasm, 29 for hiatal hernias and 41 for GERD. The mean postoperative evaluation period was of 26 months. The investigation protocol in the studied group was the performance of an esogastroduodenal barium swallow and an upper endoscopy, both pre and postoperatively. We did not have any access to pH metry or the oesophageal manometry.

Out of the 15 patients treated for achalasia (cardiospasm), we performed Heller in 14 and associated an anterior Dor hemi valve in 1 patient.

**Fig. 4:**
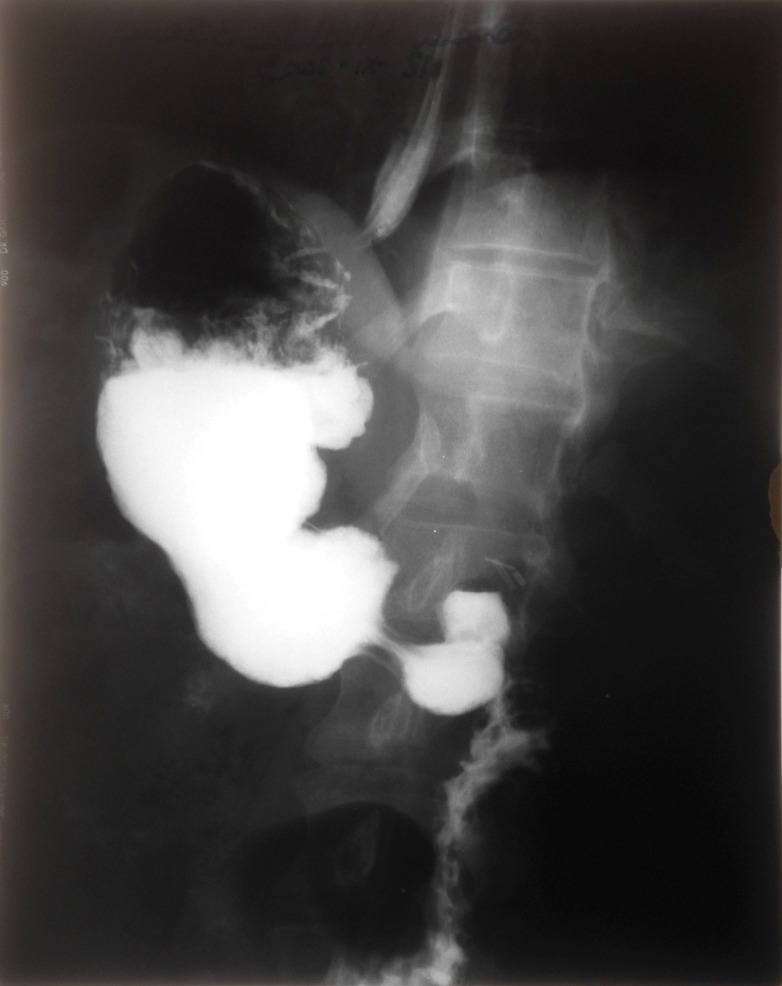
Radiological control after Nissen fundoplication (48 h postoperatively)

For the 29 patients with hiatal hernia, we performed Nissen-Rosetti in 17 cases, (**Fig. 4**) and Toupet fundoplication in 12 cases.

As far as GERD is concerned, we performed Nissen-Rosetti in 25 cases and Toupet in 17 cases.

Among the intraoperative incidents (7% of the cases) we should mention the intraoperative haemorrhage in 5 cases (5,88%), opening of the oesophageal mucosa in 1 case of achalasia (1,17%) finished by a laparoscopic suture and anterior Dor valve. There were 3 conversions to open surgery in a case of haemorrhage or of impossible adequate exposure (conversion rate was of 3,52%).

For the 29 patients with hiatal hernia, Nissen Rosetti was performed in 17 and Toupet fundoplication in 12.

### Intraoperative incidents

As described in the medical literature, Nissen fundoplication creates a zone of over competent pressure, at the LES level, from where dysphagia, one of the most frequent complications, evolves. As far as we are concerned, we had 6 cases of postoperative dysphagia after Nissen fundoplication, out of which a case of severe early dysphagia, which needed a laparoscopic intervention to undo the operation. In the other 5 cases, dysphagia was of low intensity and it resolved after a conservatory treatment, in 2-9 months. The incidence of the dysphagia in the studied group was of 24%. There was no postoperative dysphagia after Toupet. Eructation was impossible in 3 cases after Nissen and the same happened in one case after Toupet, and it passed in a few days when the postoperative oedema passed.

Hernia relapse was found in 3 patients after Nissen and in one after Toupet fundoplication; it was corrected through laparoscopic reintervention with recalibration of the oesophageal hiatus with a substitution prosthesis in 2 cases. Other complications: pneumopathy in each of the two cases after each type of intervention, and it passed under medical treatment and subcutaneous emphysema in 3 patients, which spontaneously disappeared in a few days.

The relapse of the reflux phenomena was registered in a case after Nissen fundoplication and in 2 cases after Toupet fundoplication, with the ascension of the valve in the thorax and the relapse of the hernia in which it was classically re operated on, with the repair of the hernia defect with a textile prosthesis. In case of the hernia relapse after Toupet fundoplication, it was laparoscopically re operated on, thus practicing the repair of the hernia defect and the relapse of the valve. Moreover, the medical literature reports more stable results with complete fundoplication. The postoperative results from the studied group may be classified as class 1 and 2 on the Visick scale. No perioperative mortality was registered.

## Discussions

Without any doubt, the antireflux technique with the best results and also the most durable one is Nissen fundoplication, a fact that was confirmed by the majority of the world surgeons. However, this cannot always be done, due to the intraoperative anatomy conditions or to the deficit in technique. [**9,10**].

The progressive follow up of the laparoscopic dissection and the evaluation of the anatomic particularities of the esogastric region, all lead to the choice of the type of antireflux procedure. Likewise, in the case of a short oesophagus, although mobilized and sufficiently dissected, the risk to slide into the thorax is important, and the valve may be made around the stomach and not around the oesophagus. 

Another significant problem found intraoperatively is the lack of gastric material, which, although mobilized through the sectioning of the short gastric vessels, can only be folded around the oesophagus with a risk of torsion, stenosis or tension. We used Toupet fundoplication in these cases, with excellent results.

Another problem to take into consideration is the sectioning of the short vessels. We avoided that, due to the postoperative complications and also if the gastric fundus allowed the easy sliding of the posterior valve. There were four cases in which we were obliged to recur to this technique, due to a hypertrophied and short gastro splenic ligament which did not allow the folding of the stomach around the oesophagus, without the risk of tension or splenic haemorrhage. 

Although preferred by surgeons, Nissen fundoplication has an increased incidence of mechanic complications, due to the geometry of the esogastric region, and, in our opinion, also due to error or technical difficulties. We consider both methods trustable; they assure an efficient control against reflux and also provide us with good long-term satisfaction.

## Conclusions

The functional postoperative result in patients with GERD was conditioned by the attentive rigorousness of the technical process. Paraclinical preoperative exploration must include oesophageal manometry in order to study the oesophageal motility. In patients with oesophageal functional deficits or in those without manometry, a partial Toupet fundoplication is recommended.

There were not any notable differences in our study, as far as the antireflux effects between Nissen and Toupet fundoplications are concerned. Nissen was followed by an increased incidence in the postoperative dysphagia probably caused by oesophageal motility, which was undiagnosed preoperatively through manometry.
